# Using optimal controlled singlet spin order to accurately target molecular signal in MRI and MRS

**DOI:** 10.1038/s41598-023-28425-2

**Published:** 2023-02-07

**Authors:** Jia-Xiang Xin, Guang Yang, Huojun Zhang, Jianqi Li, Caixia Fu, Jiachen Wang, Rui Tong, Yan Ren, Da-Xiu Wei, Ye-Feng Yao

**Affiliations:** 1 Physics Department & Shanghai Key Laboratory of Magnetic Resonance, School of Physics and Electronic Science, East China Normal University, North Zhongshan Road 3663, Shanghai, 200062 People’s Republic of China; 2 Department of Radiation Oncology, Shanghai Changhai Hospital, The Second Military Medical University, Shanghai, 200433 People’s Republic of China; 3 Application Developments, Siemens Shenzhen Magnetic Resonance Ltd., 518057 Shenzhen, People’s Republic of China; 4 Department of Radiology, Huashan Hospital of Fudan University, 12 Mid Wulumuqi Road, Shanghai, 200040 China

**Keywords:** Molecular neuroscience, Solution-state NMR

## Abstract

Magnetic resonance imaging (MRI) and magnetic resonance spectroscopy (MRS) have made great successes in clinical diagnosis, medical research, and neurological science. MRI provides high resolution anatomical images of tissues/organs, and MRS provides information of the functional molecules related to a specific tissue/organ. However, it is difficult for classic MRI/MRS to selectively image/probe a specific metabolite molecule other than the water or fat in tissues/organs. This greatly limits their applications on the study of the molecular mechanism(s) of metabolism and disease. Herein, we report a series of molecularly targeted MRI/MRS methods to target specific molecules. The optimal control method was used to efficiently prepare the singlet spin orders of varied multi-spin systems and in turn greatly expand the choice of the targeted molecules in the molecularly targeted MRI/MRS. Several molecules, such as *N*-acetyl-l-aspartic acid (NAA), dopamine (DA), and a tripeptide (alanine-glycine-glycine, AGG), have been used as targeted molecules for molecularly targeted MRI and MRS. We show in vivo NAA-targeted ^1^H MRS spectrum of a human brain. The high-resolution signal of NAA suggests a promising way to study important issues in molecular biology at the molecular level, e.g., measuring the local pH value of tissue in vivo, demonstrating the high potential of such methods in medicine.

## Introduction

Magnetic resonance imaging (MRI) has been widely used in clinical diagnosis, biomedical research, and neurological science^1–3^. Most MRI techniques essentially map the spatial locations/distribution of ^1^H spins of water and fat in organs^4^. Although it can image anatomy in vivo with high spatial resolution, conventional MRI techniques lack “chemical resolution” in discriminating metabolites. That is, it is difficult for MRI to selectively image/probe a specific metabolite molecule other than water and fat^1^. Complementary to MRI, magnetic resonance spectroscopy (MRS) can directly probe different metabolites^1,2,5^. A routine MRS procedure includes the acquisition of MR images and the corresponding MR spectra. Here, MR images are used to indicate the spatial location of the MR spectra. MRS can help explain the molecular mechanism of metabolism and disease and in turn facilitate accurate diagnoses, recognition, and therapy^6–9^. In clinical applications, MRS can detect diseases much earlier than anatomic MRI because abnormal metabolism often occurs long before the resulting changes in tissue/organ morphology^10,11^. MRS signals are often difficult to analyze and quantify due to signal overlap and a corrupted baseline. This originates from the complexity of the composition of metabolites and the abundance of water in the body^5,12^. Thus, selective imaging/detection of endogenous molecules in MRI/MRS will greatly facilitate their applications in clinical environments and in brain research^13–17^.

Many efforts have been made to scan specific molecules in MRI and MRS. For example, exogenous biomarkers or agents have been used to image specific molecules^16–19^. By exploiting the chemical exchange process between hydroxyl groups of water and the active hydrogens of the biological substances, Balaban et al., developed chemical exchange dependent saturation transfer (CEST)^20–22^ to indirectly detect biological substances such as amino acids and sugars. In MRS, various techniques, such as MEGA editing by Mescher and Garwood et al.^23^, double quantum filtered (MQF) MRS^23–26^, and magnetic resonance spectroscopic imaging (MRSI) by Brown et al.^27^, can selectively probe signals from specific biomolecules. Although these techniques can probe the specific molecules, they fail to accurately select a specific molecule for molecularly targeted imaging/detection.

More recently, a nuclear singlet spin state/order^28–31^ has been used to select magnetic resonance signals from a specific group and/or molecule in vitro^32–35^. For example, DeVience et al.^36^ demonstrated a method known as ‘Suppression of Undesired Chemicals using Contrast-Enhancing Singlet States (SUCCESS) to create, preserve and measure the target singlet state produces strong contrast relative to undesired spectral components when the spectra of two molecules nearly overlap. Chen et al., propose a singlet-filtered spectroscopy method based on adiabatic passage singlet-order conversion (SFS-APSOC)^33,37^, which enables targeted detection of the coupled proton groups from extensive components in vitro. Simpson et al. reported a DREAMTIME-NMR technique which provides “a molecular window” to only refocus J-coupled magnetization while all other unwanted signals^38^. Glöggler et al., demonstrated a general coupling magnetization-to-singlet (gc-M2S) singlet-filtered method^34^ to distinctly observe the glutamate (Glu) in the hippocampus of a living mouse in vivo. Numerous π hard pulses in the gc-M2S sequence may inevitably bring about the RF power deposition with the risk of biological tissues heating in human brains in vivo.

In this work, we report a series of molecularly targeted MRI/MRS methods based on optimal control (OC) of spin singlet. The OC method^33,39^ provides the high efficiency and flexibility for systematically imposing desirable constraints and fully exploits the experimentally available high degree of freedom in pulse sequences to design experiments with specific properties which are typically not covered by traditional pulse design procedures. Thus, the OC method is suitable for weakly, strongly, and multiple spin systems. Besides, an optimized control pulse is composed of hundreds of pulse units, which avoids RF power deposition from π hard pulses. Taking advantage of the precise control and high efficient preparation of singlet order by using OC pulses, we demonstrate that it is possible to target the MR signals of the specific molecules in humans in vivo. Several molecules, such as *N*-acetyl-l-aspartic acid (NAA), dopamine (DA), and a tripeptide (alanine-glycine-glycine), have been used as target molecules in molecularly targeted MRI and MRS. Moreover, the analysis of the in vivo NAA targeted ^1^H MRS spectrum of a human brain suggests a promising way to study important issues of molecular biology at the molecular level, e.g., measuring the local pH value of tissue in vivo, which is important for one to understand the molecular mechanism of biological processes^35,40,41^.

## Experiments

### Materials

All reagents were used as received without further purification including: *N*-acetyl aspartate (NAA, 99%, Sigma-Aldrich), Dopamine hydrochloride (DA, 98%, Aladdin), a tripeptide (alanine-glycine-glycine, AGG, 98.89%, Nanjing Peptide Biotech Ltd), deuterium oxide (D_2_O, 99.9%).

### Sample preparation

1. Sample 1: The AGG, NAA, and DA aqueous solutions (concentration of each solution sample, 0.08 mol per liter) were prepared by dissolving the solutes into D_2_O as follows: First, four capillary tubes (1 mm diameter, ~ 0.1 mm thickness), filled with water (80% D_2_O and 20% H_2_O), NAA, AGG, and DA aqueous solution, were prepared, respectively. Then the four capillary tubes were carefully inserted into a 5 mm NMR tube that already contains about 500 micro-liter volume water (60% D2O and 40% H2O).
2. Sample 2: Seven samples of the NAA with different pH (the pH values are 4.6, 5.4, 5.8, 6.4, 6.7, 7.4, and 7.6, respectively) were prepared. For each sample, pH was adjusted to the desired value with a calibrated pH meter (Jenway-3510, England) by adding the concentrated NaOH solution (0.05 mol per liter) and/or HCl (0.05 mol per liter).

### Subjects

Three healthy volunteers including one 25-year-old female (weight 50 kg, height 150 cm), one 24-year-old male (weight 65 kg, height 177 cm) and one 25-year-old male (Weight 63 kg, height 175 cm), were scanned for the molecularly targeted MRS. All methods were performed according to the guidelines of the Declaration of Helsinki and the current ethical guidelines. This study was approved by the Institutional Ethics Committee of East China Normal University (Shanghai, People’s Republic of China). Written informed consent was obtained from all the study participants prior to their enrolment. Informed consents were obtained from all subjects. All experiments were carried out on a 3T Siemens MAGNETOM Prisma scanner.

## Results and discussion

**Figure 1:**
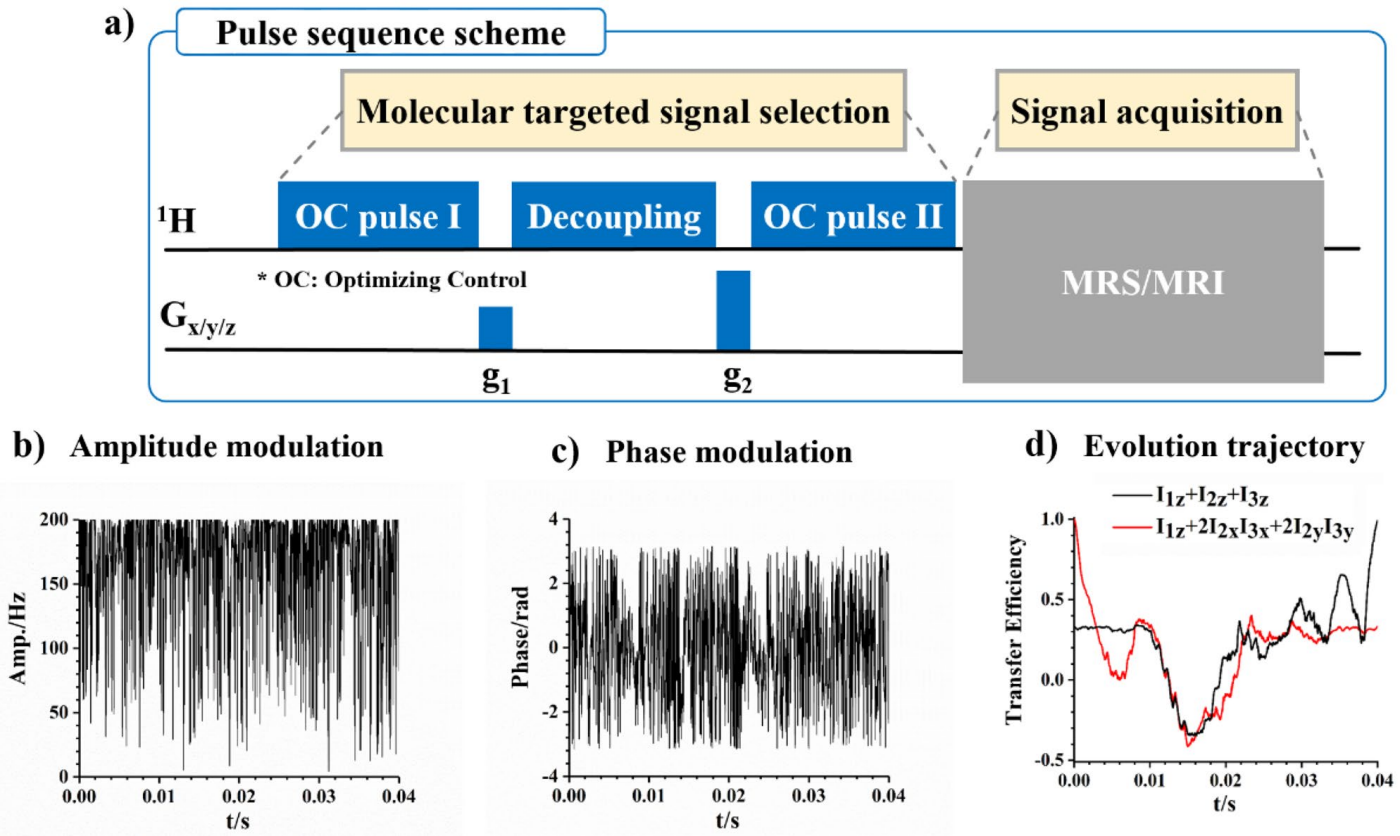
(**a**) The pulse sequence scheme of molecularly targeted MRI/MRS, namely SFOC-MRS or SFOC-MRI. Here, the solid blue rectangles represent the optimal control pulses (OC pulse I and II) and the decoupling block, respectively. The solid black rectangles, g_1_ and g_2_, are the gradient field pulses used for coherence dephasing. The grey rectangle represents the pulse sequence block for MRS/MRI. (**b**) The amplitude modulation and (**c**) the phase modulation an OC pulse as an example. (**d**) The evolution trajectory of a three-spin system under OC pulse I. The spin evolutions of the different spin systems and the specific pulse sequence examples can be found in Supplementary Materials.

Figure 1a illustrates the scheme of the molecularly targeted MRS/MRI pulse sequences. In the pulse sequence, the Singlet order Filtering method based on an Optimal Control pulse (SFOC) is used to selectively probe the signals of endogenous molecules in vivo. The sequences consist of two parts: One for molecularly targeted signal selection and one for MRI/MRS acquisition. The molecular selectivity of the pulse sequences originates from the preparation of the singlet spin order (SSO). Sequence parameters used in SSO preparation were calculated using chemical shifts and J-couplings of a spin system in the targeted molecule. In principle, any molecule with a spin system suitable for SSO preparation can be used as the target. The efficiency of SSO preparation is related to the design of the pulse sequence. Here, the OC strategy is exploited to prepare SSO. Figure 1b and c show the amplitude modulation and the phase modulation of an OC pulse as an example. Both the amplitude modulation and the phase modulation consist of a series of modulated discrete units, which are optimized to ensure the highest efficiency of SSO preparation. Because SSO is not a single quantum coherence and is not directly observable^42^, the OC pulse II is required in the pulse sequence in Fig. 1a to transfer the SSO back into longitudinal polarization. MR signal is acquired by the following MRI/MRS sequence block. Figure 1d shows, as an illustration, the spin evolution of a three-spin NAA system (H^a^, H^b^, and H^b'^) during the OC pulse I. the chemical shift and J couplings of NAA could be found in Supporting Information Fig. S1. 4000 individual short pulses (total duration: 40 ms) make up the OC pulse. The longitudinal magnetization (the black line) is transferred to the spin order (I_1z_ + 2I_2x_I_3x_ + 2I_2y_I_3y_) with the highest efficiency. Comparing with the reported singlet-filtered methods for signal selection, including gM2S^43^, SUCCESS^36^, SISTEM^35^, the main advantage of the OC pulses lies on the accurate control of the spin dynamics of a multiple spin system, resulting in the high preparation efficiency of the singlet order of the multiple spin system (see Fig. S2). Herein, some important biochemicals, such as AGG, NAA, and DA, have been successfully targeted in the molecularly targeted MRI/MRS. More details about the spin evolution of specific pulse sequence can be found in Supporting Information.

**Figure 2:**
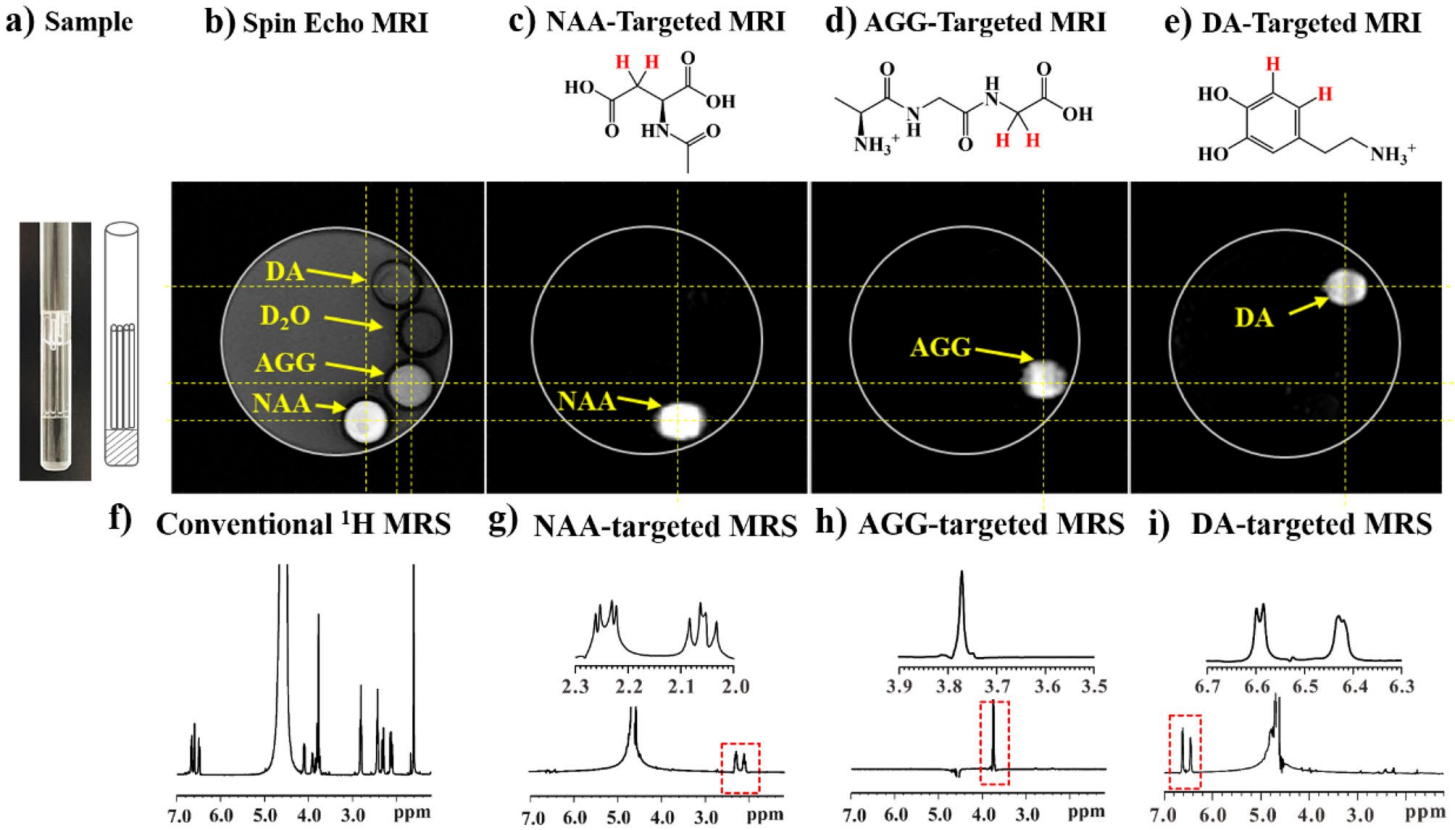
(**a**) A photo of the Sample 1. This sample is a 5 mm NMR glass tube containing water (40% D_2_O and 60% H_2_O). Four capillary tubes (1 mm diameter) containing D_2_O and the NAA, AGG, and DA aqueous solution were carefully set into the 5 mm tube. The (**b**) spin echo, (**c**) NAA-targeted, (**d**) AGG-targeted, and (**e**) DA-targeted ^1^H MRI image of the sample. The (**f**) conventional, (**g**) NAA-targeted, (**h**) AGG-targeted, and (**i**) DA-targeted ^1^H MRS spectra of the same sample. For the NAA-targeted MRI and MRS, the signals of the CH_2_ group of NAA (marked by red in the NAA molecular structure in **c**) were selected. For the AGG-targeted MRI and MRS, the signals of the CH_2_ group of AGG (marked by red in the AGG molecular structure in **d**) were selected. For the DA-targeted MRI and MRS, the signals of the two CH groups of DA (marked by red in the DA molecular structure in **e**) were selected. A ^1^H spin echo imaging sequence (see Fig. S3) was used to acquire the image in (**b**). The molecular targeted MRI images of the sample were acquired using SFOC-MRI-11.7T (see Fig. S4). A conventional MRS spectrum was acquired using the spin echo sequence (see Fig. S5). The molecularly targeted MRS spectra of the sample were acquired using SFOC-MRS-11.7T (see Fig. S6). The amplitude and phase of the OC pulses of AGG, NAA and DA used in the experiments are shown in the Figs. S7–S9. More experimental details can be found in Supporting Information. The nutation frequency of Continuous Wave (CW) decoupling pulse in SFOC-MRS-11.7T and SFOC-MRI-11.7T sequences is about 2 kHz (0.1 W for Bruker Triple resonance Broadband Inverse probe) and the duration is 200 ms. The experiment was done at room temperature. All experiments were carried out on a 500 MHz Bruker AVANCE III spectrometer.

To demonstrate the signal targeting by using the developed pulse sequences, a phantom, i.e., Sample 1 in Fig. 2a, was prepared. Figure 2b shows the routine ^1^H MRI image of Sample 1 that was acquired with a spin echo imaging sequence. The pulse sequence is referred to Fig. S3. In the routine ^1^H MRI image of Sample 1, one can see one big grey disk containing four small disks, each surrounded by a black circle representing the wall of the capillary wall. The big grey disk is from the water inside the 5 mm glass tube, whereas the smaller disks are from the capillaries containing HDO water, the NAA, AGG, and DA aqueous solutions, respectively. The difference in signal intensity can be attributed to the difference in ^1^H concentrations in different solutions. Figure 2c–e show the molecularly targeted ^1^H MRI image of NAA, AGG, and DA, respectively. These were acquired using SFOC-MRI-11.7T (see Fig. S4). The capillary containing the targeted molecule can be selectively imaged, thus demonstrating the excellent molecular selectivity of SFOC-MRI-11.7T.

Figure 2f–i shows the MRS spectra of the Sample 1. Figure 2f is the conventional ^1^H MRS spectrum, and Fig. 2g–i are the molecularly targeted MRS spectra acquired using SFOC-MRS-11.7T. The pulse sequence used to acquire conventional ^1^H MRS spectra can be found in Fig. S5. In the conventional ^1^H MRS spectrum in Fig. 2f, the signals from the HDO, NAA, AGG, and DA are clearly observable. In the molecularly targeted MRS spectra, i.e., Fig. 2g–i, acquired using SFOC-MRS-11.7T (see Fig. S6), the signals of the targeted molecules are dominant in the spectra. The other signals including the water signal are significantly suppressed, indicating the excellent molecular selectivity of the method. The life-time T_s_ of the singlet orders for the molecules was experimentally measured, which is much longer than the corresponding spin–lattice relaxation time T_1_ (see Fig. S10 for the details).

**Figure 3:**
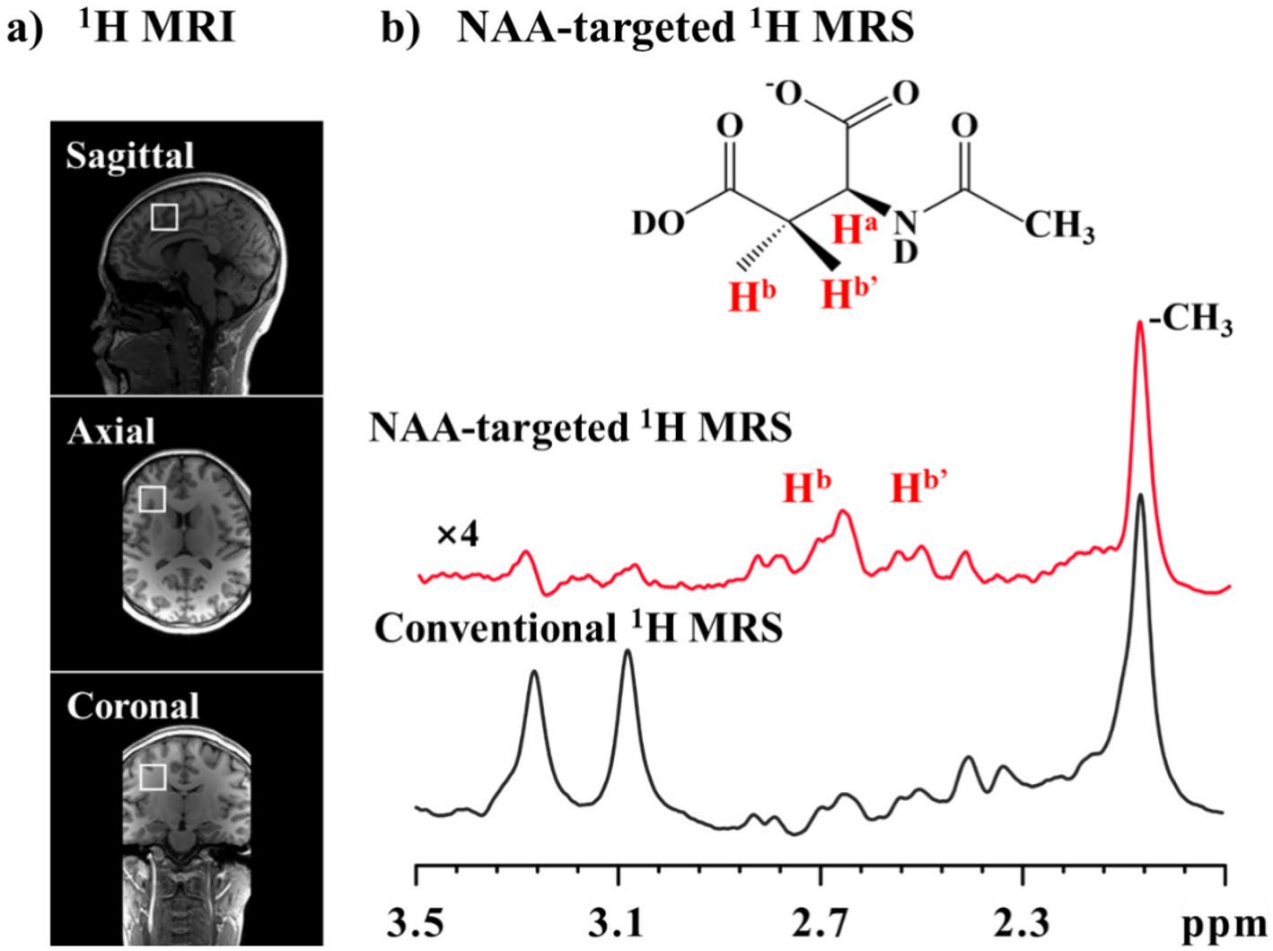
(**a**) Routine MRI images showing the voxel position in the human brain. The following ^1^H MRS spectra were acquired in this voxel with a size of 20 × 20 × 20 mm^3^; (**b**) the routine ^1^H MRS spectrum (black) and the NAA targeted ^1^H MRS spectrum (red) acquired by using SFOC-MRS-3T (see Fig. S11). The amplitude and phase of the OC pulses of NAA in the experiments are shown in the Fig. S12. The scan time of 64 scan accumulation in NAA-targeted experiments is about 3.2 min. The nutation frequency of the decoupling pulse in SFOC-MRS-3T sequence is 200 Hz (about 200 W for quadrature body RF coil) and the duration is 5 ms. More experimental details can be found in Supporting Information. The experiments were carried out on the Siemens 3T Prisma MRI scanner.

We have also used SFOC-MRS-3T (see Fig. S11) to probe the NAA molecule in human brains in vivo. The OC pulses used in the SFOC-MRS-3T are shown in Fig. S12. Figure 3a and b are the MRI image and a traditional ^1^H MRS of a human brain, respectively. The region of interest (ROI) where ^1^H MRS was acquired is marked with a white box in Fig. 3a. The MRS spectrum is featured with severe signal overlapping which can be attributed to the complex biochemical environment in the human brain. The red line in Fig. 3b presents the in vivo NAA-targeted ^1^H MRS acquired using SFOC-MRS-3T. The monitored spin system of NAA consists of H^a^, H^b^, and H^b’^ (see cartoon in Fig. 3b). The SSO of NAA (H^b^, H^b'^) was prepared in the three-spin system, and the signals of H^b^ and H^b'^ in the CH_2_ group were selected afterward. Given the maximum achievable B1 field strength and limitations on specific absorption rate (RF energy deposition), the decoupling pulse partially reserved the ^1^H signals of CH_3_ (20% routine ^1^H spectrum). The signals of endogenous NAA molecule (H^b^ and H^b’^) in NAA targeted ^1^H MRS spectrum have been observed distinctly in vivo while the signals of biomolecules such as lipids, Glutamate (Glu), Glutamine (Gln), γ-Aminobutyric acid (GABA), total Choline (choline-containing compounds, tCho) and total Creation (sum of creatine and phosphocreatine, tCr) are significantly suppressed. Analyzing the numerical integration shows that the signal intensity of NAA-CH_2_ in the NAA-targeted spectrum is ⁓ 30% of the signal intensities in the routine MRS spectrum, and the signal intensities of tCr and tCho are less than 5% of the original signal. The pulse sequence has been applied on three healthy subjects and the yielded spectra are quite similar, demonstrating the feasibility of SFOC-MRS-3T (see Fig. S13). Note that the J-coupling between the two ^1^H spin pairs, i.e., (H^a^, H^b^)_,_ and (H^a^, H^b’^), can be easily measured via the signal splitting in the spectrum. The accurate measurement of the NAA signals (i.e., the chemical shifts) provides a way to probe the pH values of the local environment of NAA, which will be discussed in detail later.

**Figure 4:**
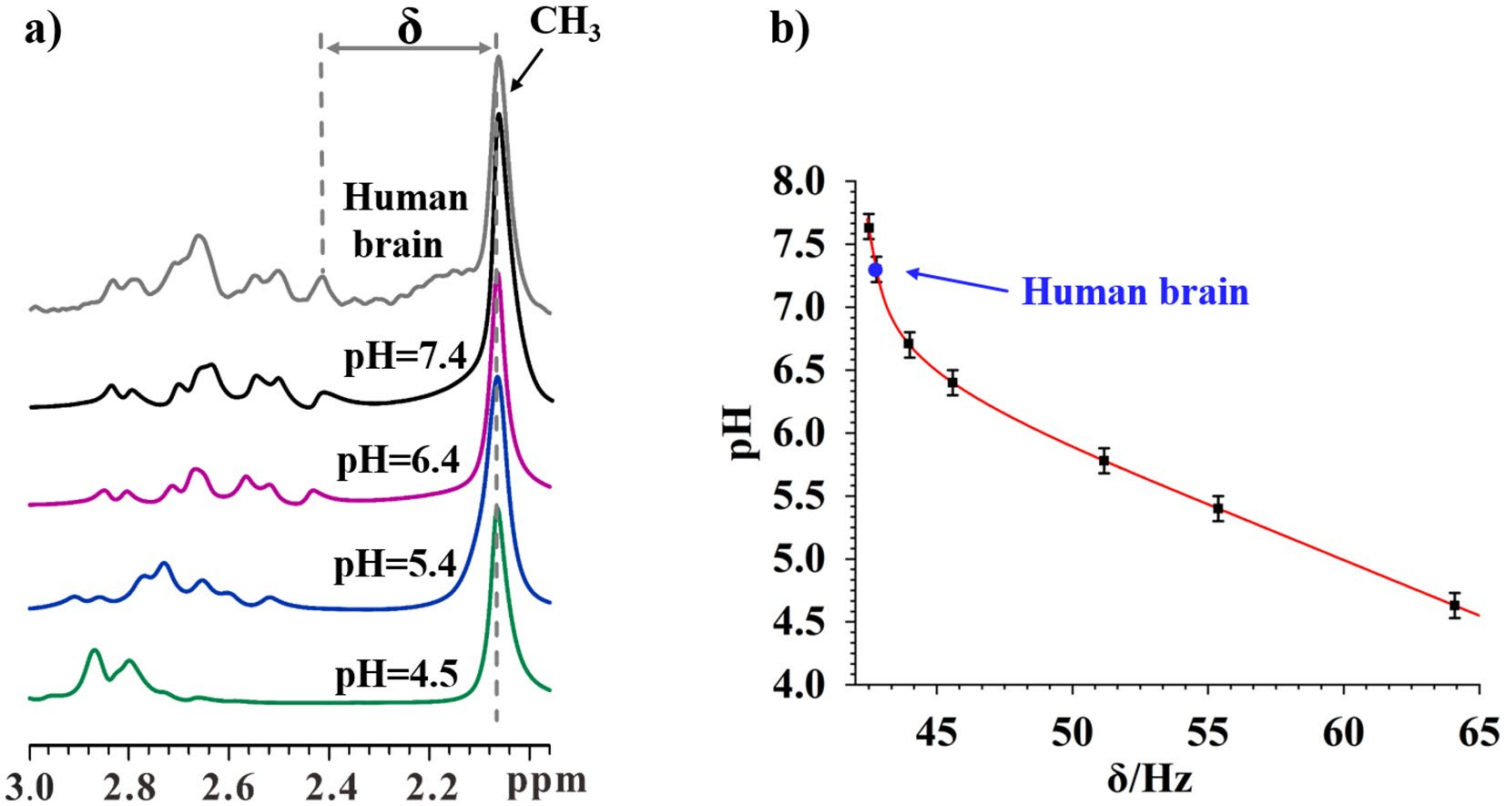
(**a**) The ^1^H MRS spectra of NAA aqueous solution containing buffers at the various pH values (black, purple, blue, and green lines) and the NAA-targeted ^1^H MRS spectrum of a human brain (the grey line); (**b**) the plot of the frequency difference between the CH_3_ signal and the first peak of H^b’^ signals of NAA (see **a**), δ, as a function of the pH values. The full width at half maximum (FWHM) of NAA signals in NAA-targeted spectrum is approximately 3 Hz. The error margin of the pH measurement using SFOC-MRS-3T sequence is ~ 0.1 when the frequency uncertainty of the NAA signals is 0.5 Hz (up limit).

Selectively probing endogenous biomolecules with MRI/MRS can have many promising applications in molecular biology and medicine. For example, it may be an easy way to measure pH. In ^1^H NMR, the chemical shift of a molecule often depends on the pH of the local environment^44^. Probing the signal of a pH sensitive molecule in vivo can thus measure the local pH values in vivo. Figure 4a shows the ^1^H MRS spectra of the NAA samples with different pH values. To facilitate this comparison, the in vivo ^1^H MRS spectrum of the human brain is also shown in Fig. 4a (grey line). Clearly, the chemical shift difference (δ) between the rightmost peak of the H^b’^ signals and the CH_3_ peak changes upon the pH value variation. The ^1^H MRS spectrum of the human brain nicely matches that from the NAA aqueous solution with a pH value of 7.4. Therefore, the NAA-targeted ^1^H MRS provides an easy way to probe the pH in human brains in vivo.

We also measured the frequency difference, δ, between the rightmost peak of the H^b’^ signals and the CH_3_ peak of NAA (see Fig. 4a) in the ^1^H MRS spectra of the NAA aqueous solution samples with different pH values. Figure 4b plots the frequency difference, δ, and the pH values. The ^1^H MRS spectra of the NAA solution samples having different pH values can be found in Figure S14 in Supporting Information. This plot is a reference curve to measure the local pH values in vivo with NAA-targeted ^1^H MRS. Note that in this plot, the frequency difference, δ, is not very sensitive to the pH values ranging from 6.8 to 7.5. However, in principle the sensitivity can be easily improved when the external field, B_0_, increases. Furthermore, this sensitivity can also be improved via alternative pH-sensitive biomolecules. This might lead to a new avenue for the in vivo pH measurement.

It is worthy of noting that B_0_ and B_1_ inhomogeneity may have influences on the performance of the OC pulse sequences. We have considered such influences during the design of the OC pulse sequence. Some simulations were performed to study the robustness of the OC pulse sequence upon the B_0_ and B_1_ inhomogeneity. Figure S15a shows the frequency offset dependence of the OC pulse. The carrier frequency was centered at the H^b’^ signals of NAA and varied between − 7.5 Hz and 7.5 Hz, which is in the range of the typical frequency variation induced by the B_0_ inhomogeneity in a 3T scanner. The simulation results show that this causes at most 5% loss of the transfer efficiency of SSO in our pulse sequence. Figure S15b shows the B_1_ amplitudes dependence of the OC pulse. The B_1_ amplitudes of OC varies from − 10% A_max_ to 10% A_max_. The simulation indicates that this causes at most 10% loss of the transfer efficiency of SSO in our pulse sequence. Based on these results, we thus conclude that B_0_ and B_1_ inhomogeneity will not cause significant influences on the performance of the OC pulse sequences in this work.

## Conclusion

In this work, based on OC of the singlet order, we developed a series of novel MR methods to implement molecularly targeted MRI and MRS. OC was used to precisely control the evolution of multiple-spin systems, and to prepare the SSOs with high preparation efficiency. Several molecules, including NAA, DA, and AGG, have been used as the targeted probes in the MRI and MRS experiments. Taking advantage of the high efficient preparation of SSOs by using OC pulses, we reported the first in vivo NAA- targeted ^1^H MRS spectra of a human brain, demonstrating the feasibility of in vivo probing of varied metabolites in humans. It is worthy of noting that the high-resolution molecularly targeted ^1^H MRS spectrum may provide many important information of the local environment of the targeted molecule. The in vivo probing of the local pH value in Fig. 4 is considered as a primary example. Compared with the other MR approaches for in vivo pH measurement such as hyperpolarized CO_2_^13^, ^31^P MRS^45^ and chemical exchange saturation transfer (CEST)^46^, the method in this work relies on the signals of the endogenous molecule (i.e., the signals of CH_2_ of NAA) and may provide a non-invasive and quick way to in vivo measure the local pH value of a living tissue. Moreover, it is expected that the high-resolution targeted ^1^H MRS spectrum also can provide the information of molecular organization in a specific local environment. For example, we found that the NAA-targeted ^1^H MRS spectrum of the healthy human brain nicely matches that from the high concentration NAA aqueous solution with a pH value of 7.4. It indicates that, although the total amount of NAA in a human brain might be low, the local concentration of NAA in a tissue most likely is very high.

We thus believe that the schemes developed in this work will have a profound influence on cell/molecular biology, brain science, medicine, pharmacology, medical physics, chemistry, physics, and MRI/NMR methodology; this might inspire many innovative ideas in the study of the chemical and physical processes in the metabolism. For example, magnetic resonance molecular imaging of biomarkers often involves using contrast agent with high specificity. Peptides, antibodies, or small ligands can be used to achieve targeting, but they must be linked to MRI contrast containing Gd or micron-sized particles of iron oxide (MPIO). This complicates the synthesis and can lead to safety concerns. With the selective imaging/probing approach shown in this work, it is possible to image specific endogenous biomarkers, thereby directly eliminating the need for exogenous contrast agents. Even when direct selective imaging of the biomarker is not possible, we can still use external targeting agents without binding them to MRI contrast agents. This can lead to new possibilities for magnetic resonance molecular imaging with implications for metabolism, early diagnosis, and drug development.

## Supplementary Information

Supplementary Information.

## Data Availability

The datasets used and/or analysed during the current study available from the corresponding author on reasonable request.
