# Supplementary material for: Using optimal controlled singlet spin order to accurately target molecular signal in MRI and MRS
Source: Sci Rep. 2023 Feb 7;13:2212. doi: 10.1038/s41598-023-28425-2 (PMC9905495; doi:10.1038/s41598-023-28425-2)
Supplement: Supplementary file 1 — Supplementary Information. [file 41598_2023_28425_MOESM1_ESM.docx]

Supplementary Materials for

Using Optimal Controlled Singlet Spin Order to Accurately Target Molecular Signal in MRI and MRS

Jia-Xiang Xin^1^, Guang Yang^1^, Huojun Zhang^2^, Jianqi Li^1^, Caixia Fu^3^, Jiachen Wang^1^, Rui Tong^1^, Yan Ren^4,*^, Da-Xiu Wei^1,*^, Ye-Feng Yao^1, *^

*Correspondence to: [dxwei@phy.ecnu.edu.cn;](mailto:dxwei@phy.ecnu.edu.cn;) [yfyao@phy.ecnu.edu.cn](mailto:yfyao@phy.ecnu.edu.cn)

This file includes:

- Magnetic resonance scanning

**Figure S1 to S15 and Table S1**

**Supplementary Text**

- Spin evolution in SFOC-MRS-11.7T

Magnetic Resonance Scanning

The experiments for Sample 1 were carried out on a 500 MHz Bruker AVANCE III spectrometer. A Bruker Triple resonance Broadband Inverse (TBI) probe equipped with a three-dimensional gradient was utilized. Experiments for Samples 2 were carried out on the 3T MRI scanner (MAGNETOM Prisma, Siemens Healthineers, Erlangen, Germany). In the Siemens 3T Prisma MRI scanner, a quadrature body RF coil and a 64-channel head-neck coil are used for transmitting the pulses and receiving the signals, respectively.


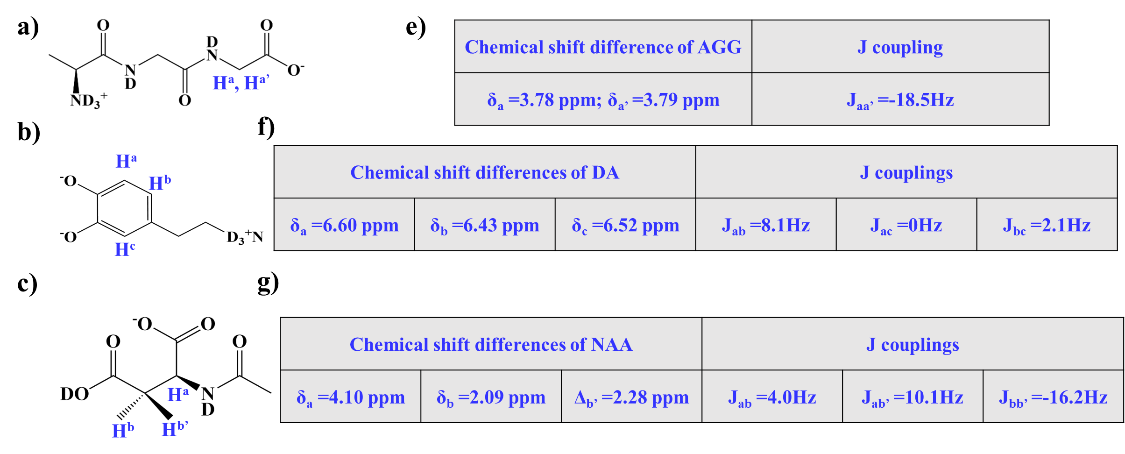


Figure S1: The molecular information of a) AGG, b) DA and c) NAA respectively. The pH values of NAA, AGG and DA were adjusted 7.3 ± 0.1.


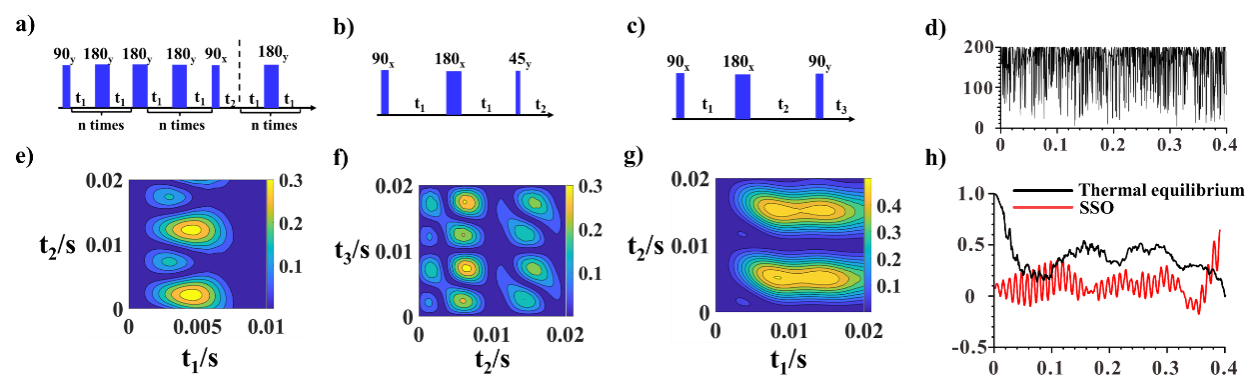


Figure S2. The sequences including a) GM2S, b) SUCCESS, c) SISTEM and d) OC pulse used to excite SSO (2I_2x_I_3x_+2I_2y_I_3y_+2I_2z_I_3z_) of NAA (H^b^, H^b’^). The simulated SSO transfer efficiency maps for NAA (H^b^, H^b’^) by using the e) GM2S, f) SUCCESS, g) SISTEM and h) OC pulse sequence. These are implemented by using the numerical calculation method in Matlab.

Table S1. The parameters used in the sequence simulation and the yielded theoretical preparation efficiency of SSO.

| **Sequences** | **Parameters** | | | **Preparation efficiency of SSO** |
| --- | --- | --- | --- | --- |
| GM2S | t_1_ = 4.6 ms | t_2_ = 2.2 ms | n = 1 | 33.50% |
| SUCCESS | t_1_ =15.6 ms | t_2_ = 6.8 ms | t_3_ = 7.4 ms | 31.70% |
| SISTEM | t_1_ = 8.4 ms | t_2_ = 5.2 ms |  | 50.10% |
| OC | The phase and amplitude of OC pulse are showed in Figure S8 | | | 66.60% |


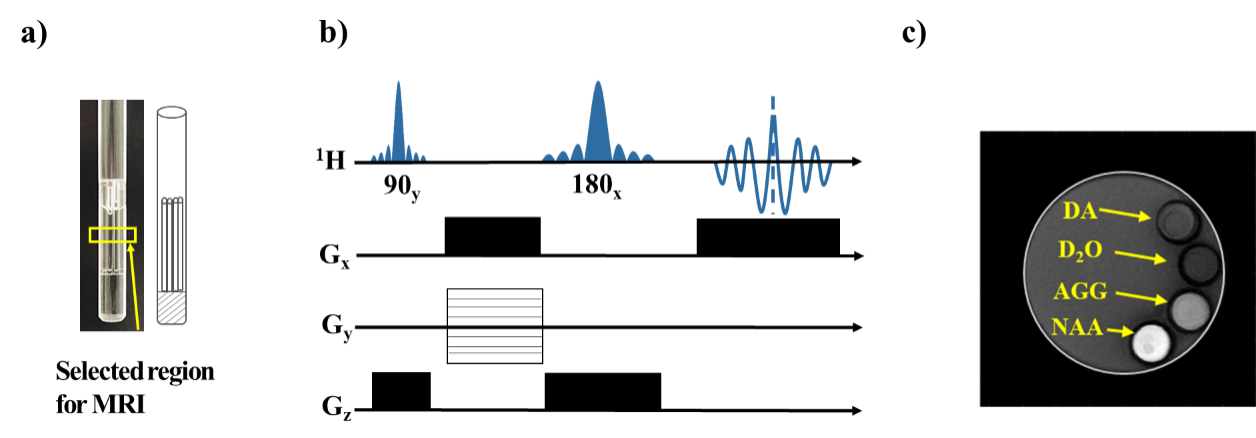


Figure S3. a) A photo of Sample 1. Yellow rectangular box represents the selected region for MRI experiments. b) The proton density-weighted MRI sequence used to acquire the image in c). The black rectangles and the blue sinc waves represent the gradient pulses and the selective pulses, respectively. c) The ^1^H MRI image of Sample 1. It can be observed that one big gray disk containing four small disks. Each surrounded by a black circle representing the wall of the capillary wall. The signal in the big gray one comes from the water inside the 5 mm glass tube. The signals in the black, gray and white disks are from the capillaries containing HDO water, the NAA, AGG, and DA aqueous solutions. The experiments were carried out on a 500MHz Bruker AVANCE III spectrometer. The following parameters of proton density-weighted MRI sequence were used: FOV = 5.8 × 5.8 mm^2^, matrix 128 ×128, in-plane resolution = 45 × 45 μm^2^, slice thickness = 0.55 cm, TR = 4.5 s, TE = 9.6 ms.


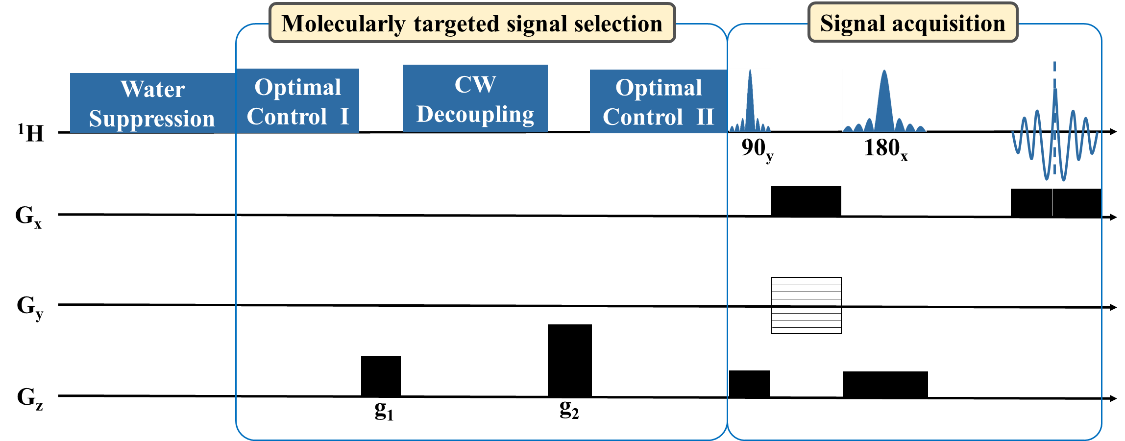


Figure S4. The SFOC-MRI pulse sequence used on the 500 MHz Bruker AVANCE III spectrometer, named SFOC-MRI-11.7T, which usually consists of three blocks. In the first block, the pre-saturation scheme is used for the water suppression. The second block is the molecularly targeted signal selection module. Three blue filled rectangles represent OC pulse I, OC pulse II, and the decoupling pulse, respectively. The black filled rectangles, g_1_ and g_2_, are the gradient pulses used for the coherence dephasing. The signal acquisition block, two sinc pulses (blue) and two gradient pulses (black) along the z direction are used to select a specific spatial position. Two gradient pulses (black) along the x direction and gradients (black) along the y direction are used to phase encoding and frequency encoding, respectively. The SFOC-MRI-11.7T sequence was applied to Sample 1. The following parameters were used: FOV = 5.8 × 5.8 mm^2^, matrix 128 × 128, in-plane resolution = 45 × 45 μm^2^, slice thickness = 0.55 cm, TR = 4.5 s, TE = 9.6 ms. Water suppression was executed in the experiments by using the pre-saturation scheme^1^ with suppression bandwidth of 35 Hz. The strength of the first and the second gradients are 5 Gs/cm, and 10 Gs/cm, respectively. The durations are 1.5 ms, 3 ms, respectively. The nutation frequency of Continuous Wave (CW) decoupling pulse is 2 kHz and the duration is 200 ms.


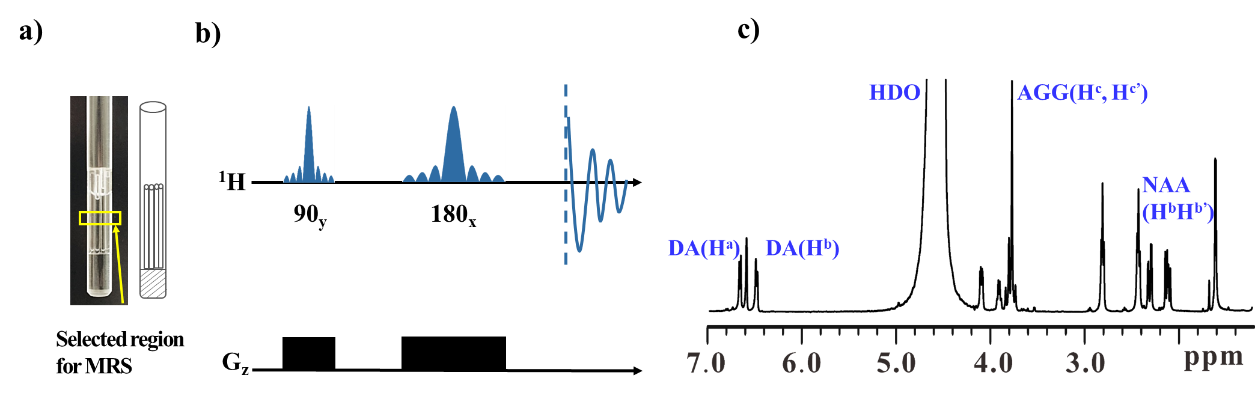


Figure S5. a) A photo of Sample 1. The yellow rectangular box represents the selected region for MRS experiments. b) The spin-echo sequence. The black rectangles and the blue sinc waves represent the gradient pulses and selective pulses, respectively. c) The ^1^H spectrum of the selected region of Sample 1. The signals from the HDO, AGG, NAA and DA can be observed in the spectrum. The low resolution and poor line shape of the signals can be attributed to the high inhomogeneity of the field due to the disturbance of the inserted capillary tubes. The experiments were carried out on a 500MHz Bruker AVANCE III spectrometer. The spin-echo sequence (Figure 5b) was applied to Sample 1. The following parameters were used: slice thickness = 0.55 cm, TR = 4.5 s, TE = 9.6 ms, averages 128, points 2048, spectral bandwidth 5000 Hz.

**
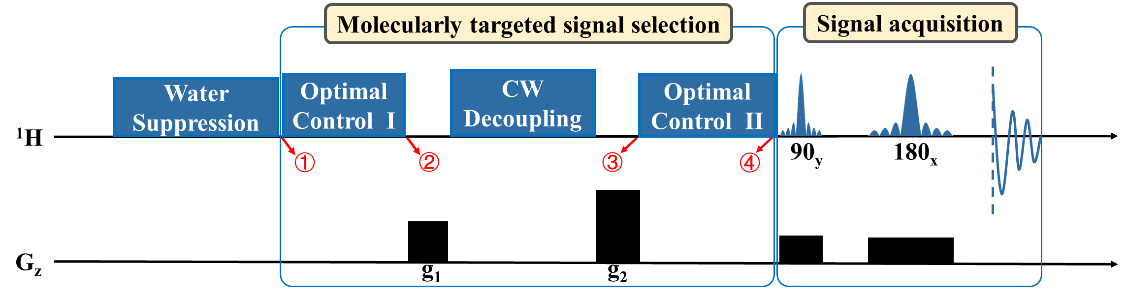
**

Figure S6. The SFOC-MRS sequence used on the 500MHz Bruker AVANCE III spectrometer, named SFOC-MRS-11.7T. Compared with SFOC-MRI-11.7T in Figure S4, the only change lies on the signal acquisition block, where the spin-echo sequence is used to acquire the signals. The SFOC-MRS-11.7T sequence was applied to sample 1. The following parameters were used: slice thickness = 0.55 cm, TR = 4.5 s, TE = 9.6 ms, averages 128, points 2048, spectral bandwidth 5000 Hz. Water suppression was executed by using the pre-saturation scheme with suppression bandwidth of 35 Hz. The strength of the first and the second gradients are 5 Gs/cm, and 10 Gs/cm, respectively. The durations are 1.5 ms, 3 ms, respectively. The nutation frequency of Continuous Wave (CW) decoupling pulse is 2 kHz and the duration is 200 ms. The remarks (①, ②, ③, ④) in SFOC-MRS-11.7T sequence were used in the density operator evolution in the following Supplementary text.

**Supplementary Text**

Spin evolution in the SFOC-MRS-11.7T sequence

The core part for signal selection in these pulse sequences are the same. The relevant product operators of each step in the core part for signal selection are given in the following.

- Prepare a SSO in a two-spin system using SFOC-MRS-11.7T

The two-spin system could be the nuclei H^a^, H^a’^ of AGG. Basing on the SFOC-MRS-11.7T sequence, the density operator evolution process of the system is:


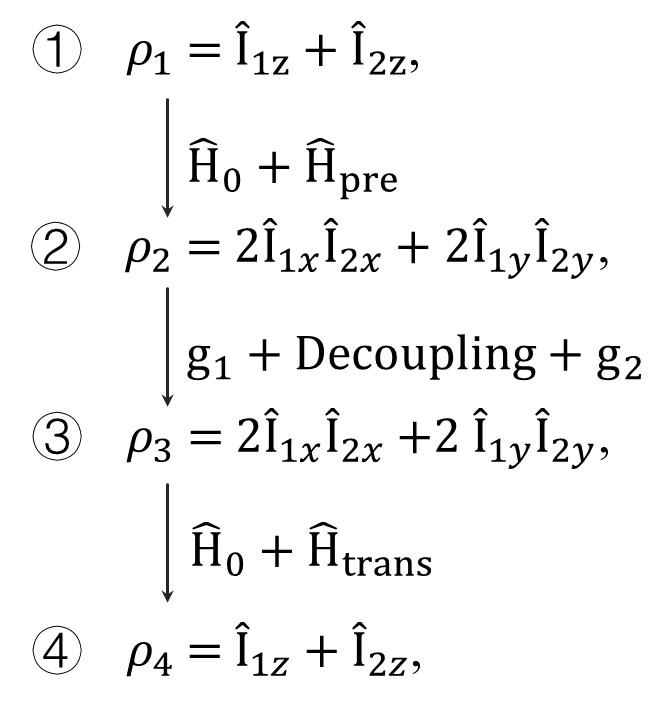


Where,

$\hat{H}_{0}=-\omega_{1}\hat{I}_{1z}-\omega_{2}\hat{I}_{2z}+2\pi J_{12}\hat{I}_{1}\cdot\hat{I}_{2}$,

$\hat{H}_{\mathrm{pre}}=2\pi P_{\mathrm{pre}}\left[ \cos\varphi_{\mathrm{pre}}(\hat{I}_{1x}+\hat{I}_{2x})+\sin\varphi_{\mathrm{pre}}(\hat{I}_{1y}+\hat{I}_{2y}) \right]$,

$\hat{H}_{\mathrm{trans}}=2\pi P_{\mathrm{trans}}\left[ \cos\varphi_{\mathrm{trans}}(\hat{I}_{1x}+\hat{I}_{2x})+\sin\varphi_{\mathrm{trans}}(\hat{I}_{1y}+\hat{I}_{2y}) \right]$.

Here, the Hamiltonian H_0_, H_pre_ and H_trans_ are in the rotation frame. J_12_ is the scalar J-coupling constant. The ω_1_ and ω_2_ are the relative Larmor frequency:

ω_1_ = ω_01_ – ω_ref_ , ω_2_ = ω_02_ – ω_ref_

ω_01_ and ω_02_ are the Larmor frequency and ω_ref_ is the frame. $\varphi_{\mathrm{pre}}$, $P_{\mathrm{pre}}$ are the phase and amplitude of the OC pulse I for preparing the nuclear spin singlet order. $\varphi_{\mathrm{trans}}$, $P_{\mathrm{trans}}$ are the phase and amplitude of the OC pulse II to transfer the nuclear spin singlet order into the longitudinal polarizations. The gradient I was used to dephase the signals from the other molecules such as H_2_O, Glutamate, Glutamine, GABA and so on. During the spin evolution process, the spin relaxation is ignored.

- Prepare a SSO in a three-spin system using SFOC-MRS-11.7T

The three-spin system could be the nuclei H^a^, H^b^, H^b’^ of NAA, or H^a^, H^b^, H^c^ of DA (denote the nuclei as I_1_, I_2_, I_3_). Basing on the SFOC-MRS-11.7T sequence, the density operator evolution process of the system is:


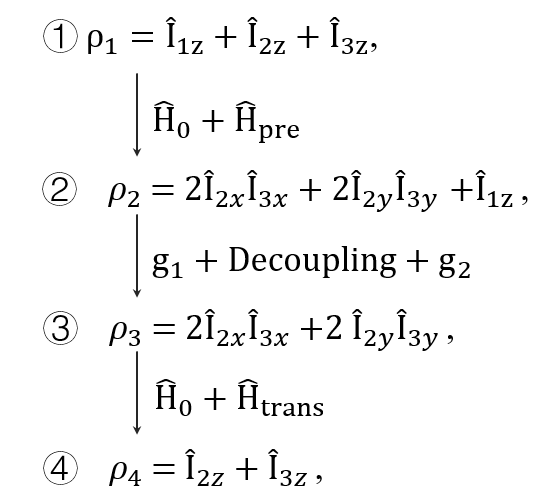


where,

$\hat{H}_{0}=-\omega_{1}\hat{I}_{1z}-\omega_{2}\hat{I}_{2z}-\omega_{3}\hat{I}_{3z}+2\pi{(J}_{12}\hat{I}_{1}\cdot\hat{I}_{2}+J_{13}\hat{I}_{1}\cdot\hat{I}_{3}+J_{23}\hat{I}_{2}\cdot\hat{I}_{3}),$

$$\hat{H}_{\mathrm{pre}}=2\pi P_{\mathrm{pre}}\left[ \cos\varphi_{\mathrm{pre}}(\hat{I}_{1x}+\hat{I}_{2x}+\hat{I}_{3x})+\sin\varphi_{\mathrm{pre}}(\hat{I}_{1y}+\hat{I}_{2y}+\hat{I}_{3y}) \right],$$

$$\hat{H}_{\mathrm{trans}}=2\pi P_{\mathrm{trans}}\left[ \cos\varphi_{\mathrm{trans}}(\hat{I}_{1x}+\hat{I}_{2x}+\hat{I}_{3x})+\sin\varphi_{\mathrm{trans}}(\hat{I}_{1y}+\hat{I}_{2y}+\hat{I}_{3y}) \right].$$

Here, the Hamiltonian H_0_, H_pre_ and H_trans_ are in the rotation frame. J_12_, J_13_, J_23_ are the scalar J-coupling constants. The ω_1_ and ω_2_ are the relative Larmor frequency of NAA (H^a^, H^b^, H^b′^) or DA (H^a^, H^b^, H^c^) :

ω_1_ = ω_01_ – ω_ref_ , ω_2_ = ω_02_ – ω_ref_, ω_3_ = ω_03_ – ω_ref_

ω_01_, ω_02_ and ω_03_ are the Larmor frequency and ω_ref_ is the frame. $\varphi_{\mathrm{pre}}$and $P_{\mathrm{pre}}$ are the phase and amplitude of the OC pulse I for preparing the nuclear spin singlet order in NAA (H^a^, H^b^, H^b’^) or DA (H^c^, H^a^, H^b^). $\varphi_{\mathrm{trans}}$ and $P_{\mathrm{trans}}$ are the phase and amplitude of OC pulse II to transfer the nuclear spin singlet order into the longitudinal polarizations. During the spin evolution process, the spin relaxation is ignored.

It should be noted that in the three-spin system, a singlet order for the spins I_2_ and I_3_ are prepared by the OC pulse I, while the polarization of the third spin is transferred to I_1z_ and then will be saturated by the decoupling pulse. The OC pulse II would change the singlet order of spins I_2_ and I_3_ into the longitudinal polarization and keep the third spin I_1_ decoupled at the same time.


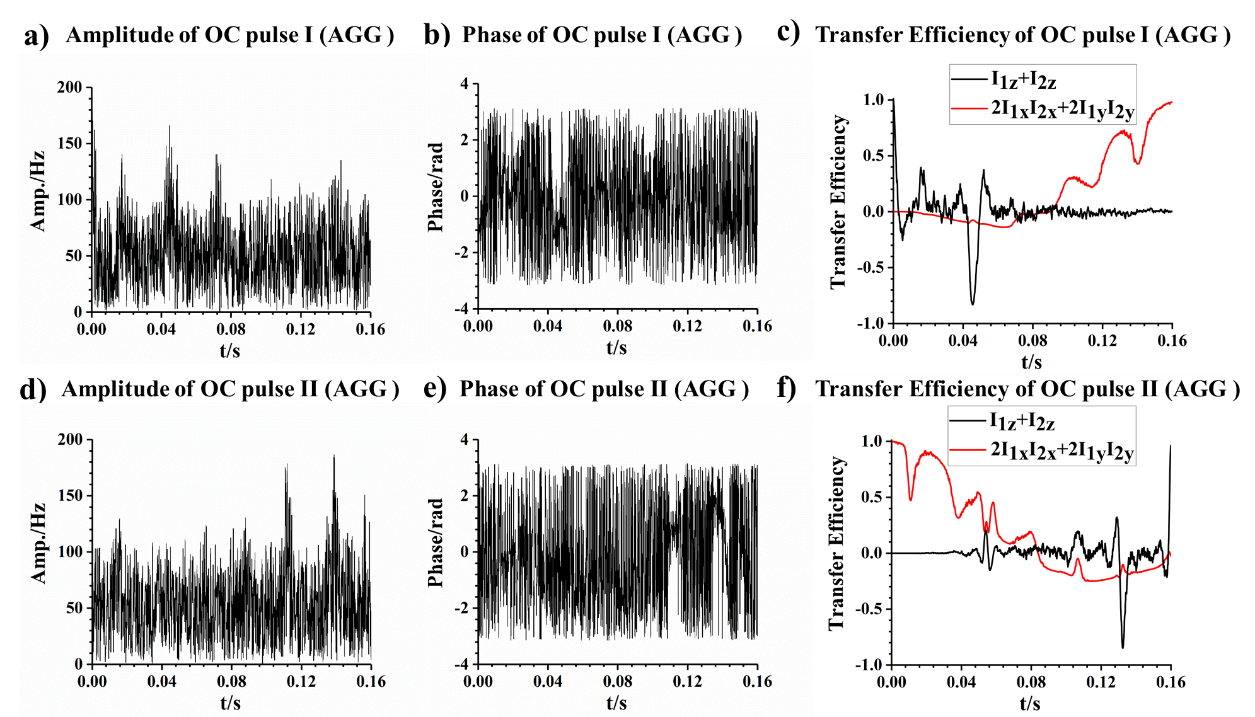


Figure S7. a) The phase and b) the amplitude of OC pulse I, and c) the evolution trajectory of the spin states operated by OC pulse I, showing that the longitudinal magnetization (the black line) is transferred to the spin order (2I_1x_I_2x_+2I_1y_I_2y_, the red line) of AGG (H^a^, H^a’^) which is composed of the singlet order and the triplet order. d) The phase and e) the amplitude of OC pulse II, and f) the evolution trajectory of the spin states operated by OC pulse II, showing that the spin order (2I_1x_I_2x_+2I_1y_I_2y_, the red line) is transferred to the longitudinal magnetization (the black line). Each OC pulse consists of 5000 individual short pulses and each individual short pulse has its specific phase and amplitude, which are defined in a profile. The total duration of 5000 individual short pulses is 160 ms. The carrier frequency of the OC pulses designed for AGG (H^a^, H^a’^) was set to 3.8 ppm (i.e., the chemical shift of H^a’^). These pulse sequences were designed for AGG (H^a^, H^a’^) in the 11.7 T B_0_ field.


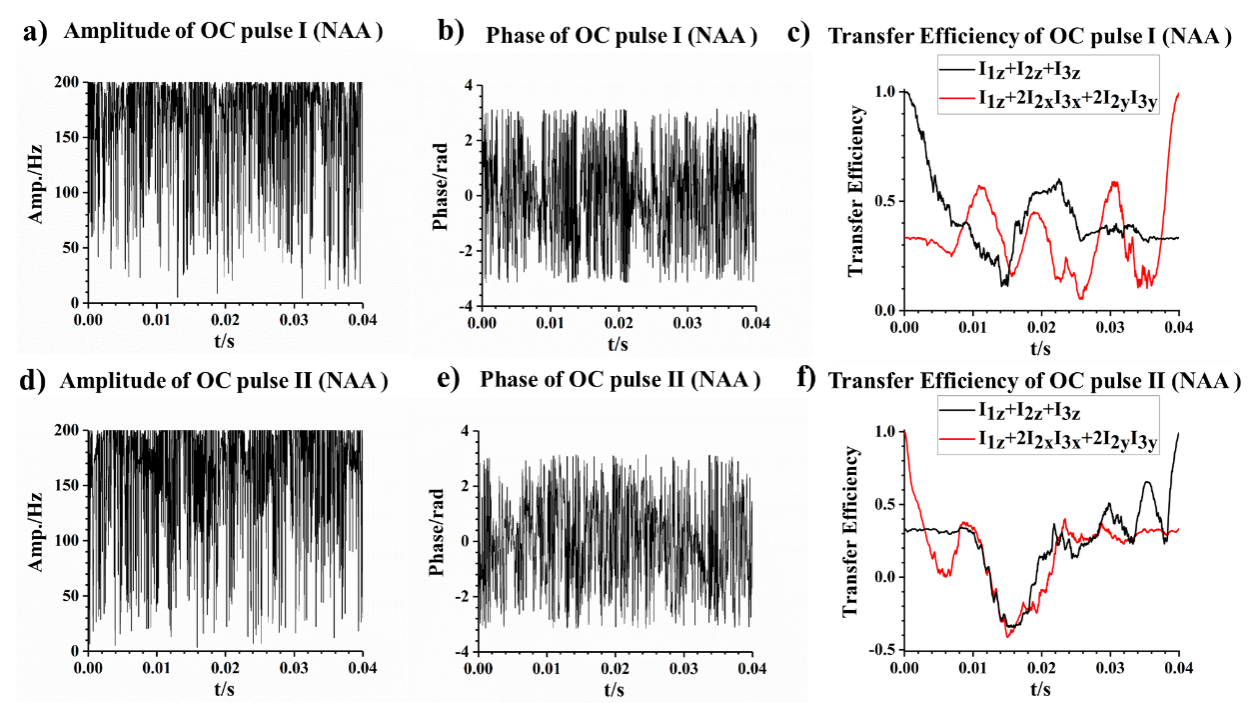


Figure S8. a) The phase and b) the amplitude of OC pulse I, and c) the evolution trajectory of the spin states operated by OC pulse I, showing that the longitudinal magnetization (the black line) is transferred to the spin order (I_1z_+2I_2x_I_3x_+2I_2y_I_3y_, the red line) of NAA (H^b^, H^b’^) which is composed of the singlet order and the triplet order. d) The phase and e) the amplitude of OC pulse II, and f) the evolution trajectory of the spin states operated by OC pulse II, showing that the spin order (I_1z_+2I_2x_I_3x_+2I_2y_I_3y_, the red line) is transferred to the longitudinal magnetization (the black line). Each OC pulse consists of 4000 individual short pulses and each individual short pulse has its specific phase and amplitude, which are defined in a profile. The total duration of 4000 individual short pulses is 40 ms. The carrier frequency of the OC pulses designed for NAA (H^b^, H^b’^) was set to 2.1 ppm (i.e., the chemical shift of H^b’^). These pulse sequences were designed for NAA (H^b^, H^b’^) in the 11.7 T B_0_ field.


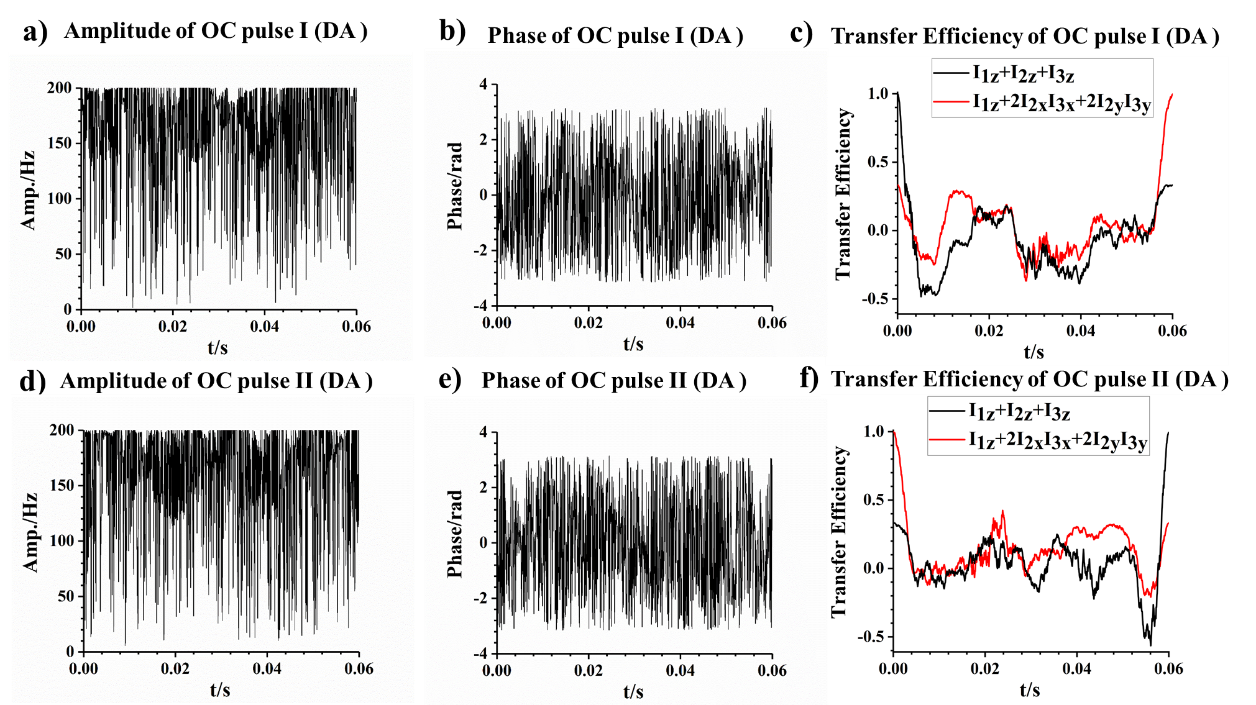


Figure S9. a) The phase and b) the amplitude of OC pulse I, and c) the evolution trajectory of the spin states operated by OC pulse I, showing that the longitudinal magnetization (the black line) is transferred to the spin order (I_1z_+2I_2x_I_3x_+2I_2y_I_3y_, the red line) of DA (H^c^, H^a^, H^b^) which is composed of the singlet order and the triplet order. d) The phase and e) the amplitude of OC pulse II, and f) the evolution trajectory of the spin states operated by OC pulse II, showing that the spin order (I_1z_+2I_2x_I_3x_+2I_2y_I_3y_, the red line) is transferred to the longitudinal magnetization (the black line). Each OC pulse consists of 4000 individual short pulses and each individual short pulse has its specific phase and amplitude, which are defined in a profile. The total duration of 4000 individual short pulses is 60 ms. The carrier frequency of the OC pulses designed for DA (H^a^, H^b^) was set to 6.45 ppm (i.e., the chemical shift of H^b^). These pulse sequences were designed for DA (H^a^, H^b^) in the 11.7 T B_0_ field.


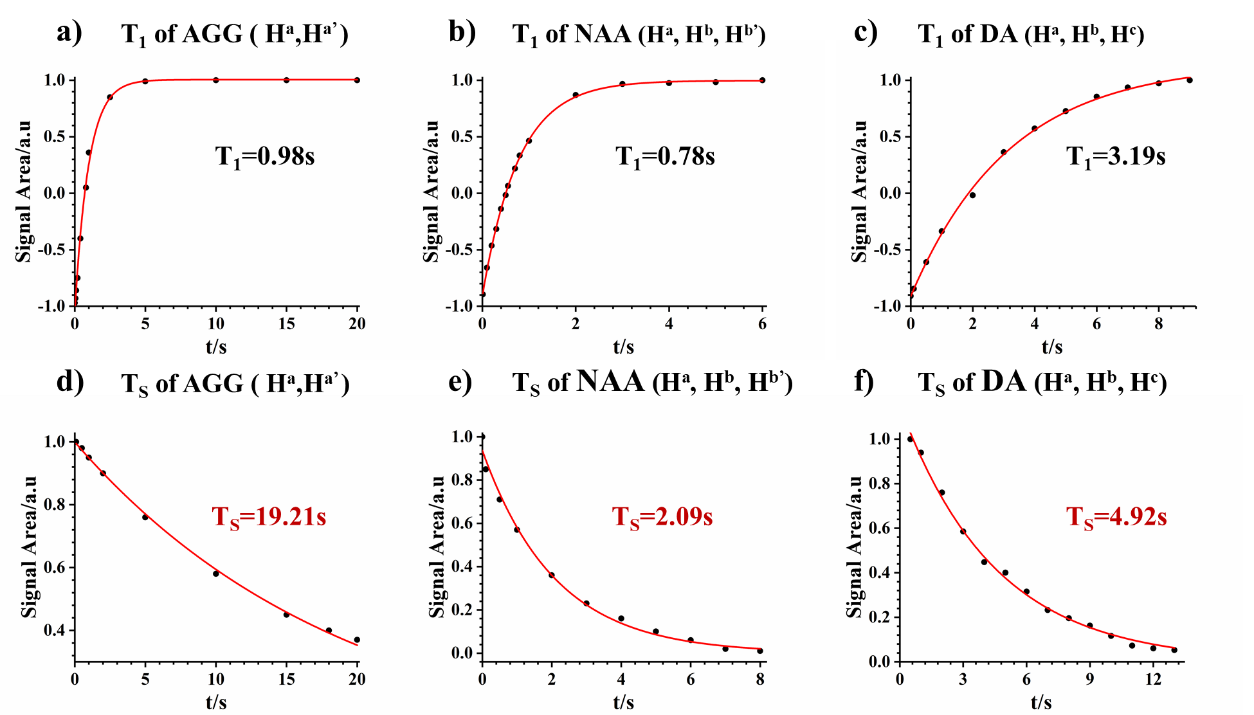


Figure S10. The T_1_ decay curves of a) AGG, b) NAA and c) DA. The decay curves of the singlet spin order of a) AGG, b) NAA and c) DA. T_1_ was measured by the inversion recovery pulse sequence. The decay curves of the singlet spin order was measured by varying the duration of the Continuous Wave (CW) decoupling pulse in SFOC-MRS-11.7T. All experiments were carried out on a 500 MHz Bruker AVANCE III spectrometer. The experimental temperature is room temperature.


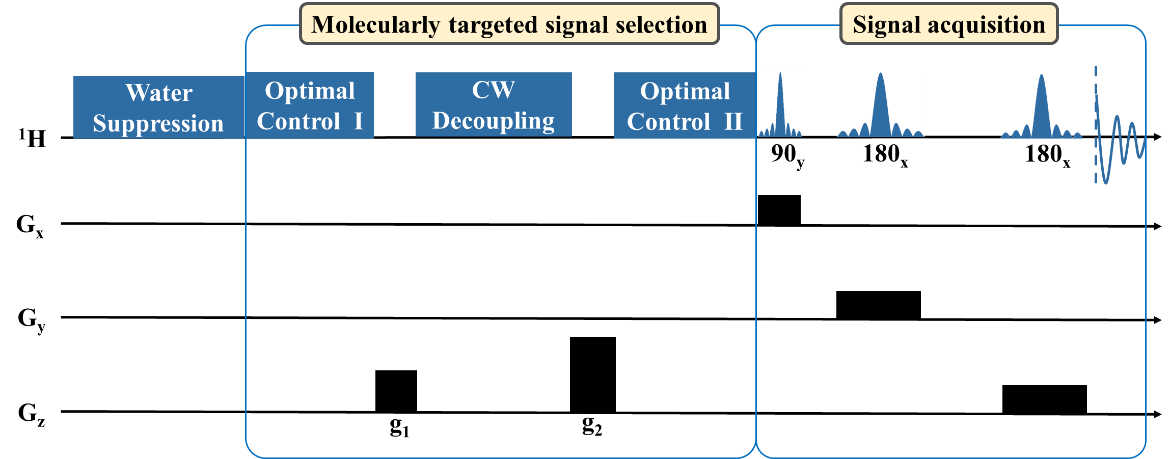


Figure S11. The SFOC-MRS pulse sequence used on a 3T Siemens MAGNETOM Prisma scanner, named SFOC-MRS-11.7T. This sequence is slightly adjusted to fit the requirement for the scanner. In the signal acquisition block, three sinc pulses (blue) and gradient pulses (black) along x, y, z direction are used to select a specific single voxel. The durations of both OC pulse I and II designed for NAA (H^a^, H^b^, H^b’^) in the sequence are 40 ms. The following parameters of the sequence were used: TR = 3.0 s, TE = 35 ms, averages 64, points 1024, spectral bandwidth 1000 Hz. The ^1^H MRS spectra were acquired with the voxel size of 20 × 20 × 20 mm^3^ in the subjects and 10 × 30 × 10 mm^3^ in the sample 2. The gradient strength is 2 Gs/cm, and the duration is 2 ms for both of two gradients. The nutation frequency of Continuous Wave (CW) decoupling pulse is 200 Hz and the duration is 5 ms. Water suppression was executed by the water suppression enhanced through T_1_ effects (WET)^2^ scheme with a suppression bandwidth of 50 Hz. The scan time of 64 scan accumulation is about 3.2 min. The acquisition time is similar to the routine sequence such as PRESS and STEAM sequences. The SAR of the sequence is 0.3 W/kg and the SAR of the conventional MRS is 0.15 W/kg. All experiments of SFOC-MRS-3T sequence were acquired on a 3T Siemens MAGNETOM Prisma scanner.


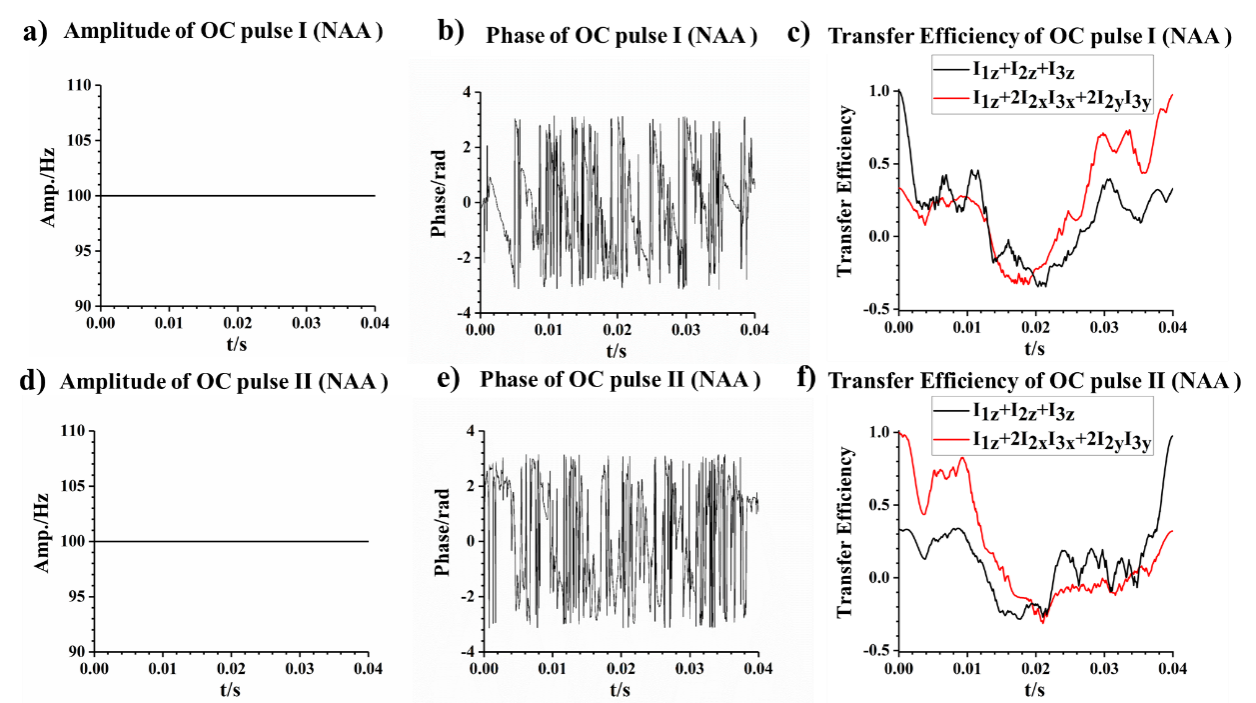


Figure S12. a) The phase and b) the amplitude of OC pulse I, and c) the evolution trajectory of the spin states operated by OC pulse I, showing that the longitudinal magnetization (the black line) is transferred to the spin order (I_1z_+2I_2x_I_3x_+2I_2y_I_3y_, the red line) of NAA (H^b^, H^b’^) which is composed of the singlet order and the triplet order. d) The phase and e) the amplitude of OC pulse II, and f) the evolution trajectory of the spin states operated by OC pulse II, showing that the spin order (2I_1x_I_2x_+2I_1y_I_2y_, the red line) is transferred to the longitudinal magnetization (the black line). Each OC pulse consists of 1000 individual short pulses and each individual short pulse has its specific phase and amplitude, which are defined in a profile. The total duration of 1000 individual short pulses is 40 ms. The carrier frequency of the OC pulses designed for NAA (H^b^, H^b’^) was set to 2.4 ppm (i.e., the chemical shift of H^b′^). These pulse sequences were designed for NAA (H^b^, H^b’^) in the 3T B_0_ field (3T Prisma, Siemens).


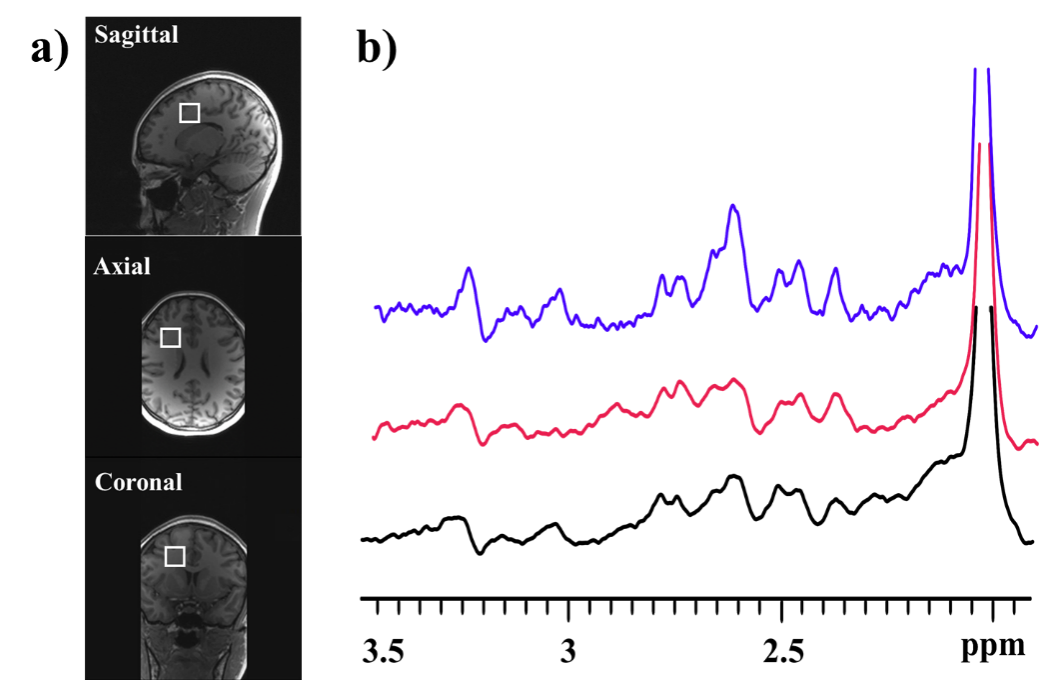


Figure S13. a) Axial, sagittal, and coronal MR images of a human brain, with depiction of the size, location, and angulation of the voxel of interest in the human brain. The MR images were acquired by using the routine T_1_-weighted MRI sequence. The following parameters were used: FOV = 200 × 200 mm^2^, matrix size = 256 × 256, in-plane resolution = 0.78 × 0.78 mm^2^, slice thickness = 2 mm, TR = 3.0 s, TE = 33 ms. b) Probing of NAA in three human brains in vivo by using SFOC-MRS-3T. Three MRS spectra are demonstrated here. These spectra were acquired by using SFOC-MRS-3T from a 25-years-old female (blue), a 24-years-old male (red) and a 25-years-old male (black). The parameters of SFOC-MRS-3T sequence can be found in Figure S11. All experiments were acquired on a 3T Siemens MAGNETOM Prisma scanner.

The pH values of all three subjects are 7.4 ± 0.1, 7.4 ± 0.1 and 7.3 ± 0.1, respectively. The average and standard deviation for the pH measurements among the three brains are about 7.4 and 0.1.


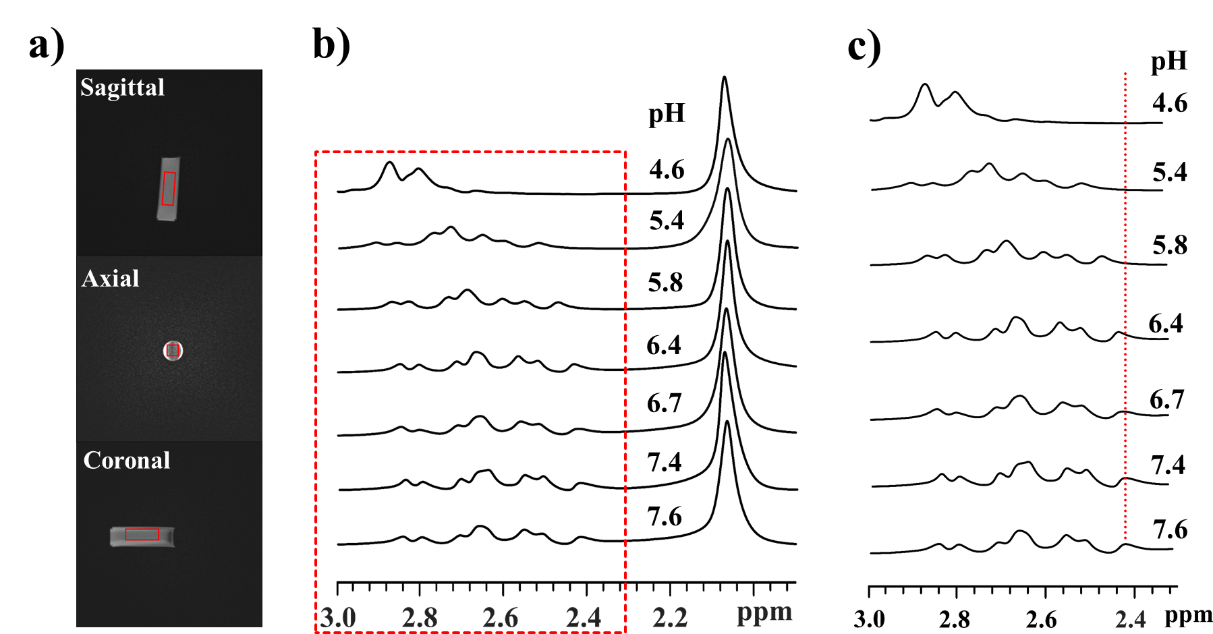


Figure S14. a) Axial, sagittal, and coronal MR images of Sample 3, with depiction of the size, location, and angulation of the voxel (10 mm × 30 mm × 10 mm) of interest in Sample 4. The MR images were acquired by using the routine multi-slice localizer sequence. The following parameters were used: FOV = 200 × 200 mm^2^, matrix size = 128 × 128, in-plane resolution = 1.56 × 1.56 mm^2^, TR = 8.6 ms, TE = 4.0 ms. b) The MR spectra of the NAA solution having different pH values by using the standard single voxel Point RESolved Spectroscopy (PRESS)^3^ sequence. The following parameters were used: TR = 3.0 s, TE = 35 ms, averages 64, points 1024, spectral bandwidth 1000 Hz. The ^1^H MRS spectra were acquired with the voxel size of 10 × 30 × 10 mm^3^ in the sample 2. Water suppression was executed by using WET scheme with suppression bandwidth of 50 Hz. c) The enlarged areas in the spectra, demonstrating the pH dependence of the signals. These experiments were acquired on a 3T Siemens MAGNETOM Prisma scanner.


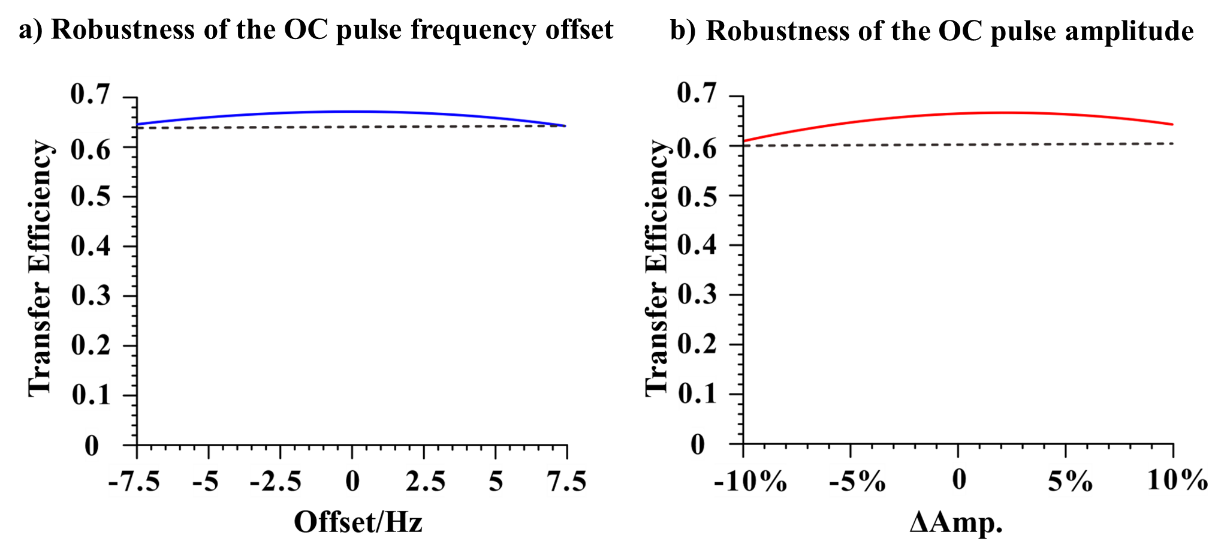


Figure S15. a) The frequency offset and b) B_1_ amplitude dependence of the efficiency transferring the longitudinal magnetization to the singlet order by using the OC pulse designed for NAA (H^a^, H^b^, H^b’^) in the simulation.

**Simulation codes of SIMPSON**

spinsys {

channels 1H

nuclei 1H 1H 1H

shift 1 0 0 0 0 0 0

shift 2 22.5 0 0 0 0 0

shift 3 233 0 0 0 0 0

jcoupling 1 2 -16.2 0 0 0 0 0

jcoupling 1 3 10.1 0 0 0 0 0

jcoupling 2 3 4.0 0 0 0 0 0

}

par {

# set up liquid state

spin_rate 0

crystal_file alpha0beta90

gamma_angles 1

start_operator I1z+I2z+I3z

detect_operator 2*I1x*I2x+2*I1y*I2y+I3z

proton_frequency 500e6

variable lam 0

variable NOC 1000

variable duration 50000

variable SWH 10

variable cp 10

sw 40000

oc_tol_cg 1e-4

oc_tol_ls 1e-3

oc_mnbrak_step 10

oc_max_iter 700

conjugate_fid false

}

proc pulseq {} {

global par rfsh

reset

pulse_shaped $par(duration) $rfsh

oc_acq_hermit

}

proc get_lims {SWH cp} {

if { $cp <= 1} {

set Res 0

} else {

set step [expr $SWH/($cp-1)]

set Res {}

for {set i 0} {$i < $cp} {incr i} {

set shft [expr $SWH/2.0-$i*$step]

lappend Res $shft

}

}

return $Res

}

proc gradient {} {

global par lims rfsh

set par(np) $par(NOC)

set f [fcreate -np $par(NOC) -sw $par(sw) ]

foreach shft $lims {

set g [fsimpson [list [list shift_1_iso $shft]]]

fadd $f $g

funload $g

}

oc_grad_add_energy_penalty $f $rfsh -$par(lam)

return $f

}

proc target_function {} {

global par lims rfsh

set par(np) 1

set Res 0.0

foreach shft $lims {

set f [fsimpson [list [list shift_1_iso $shft]]]

set dum [findex $f 1 -re]

set Res [expr $Res+$dum]

funload $f

}

set en [shape_energy $rfsh $par(duration)]

set Res [expr $Res - $par(lam)*$en]

return [format "%.20f" $Res]

}

proc main {} {

global par rfsh lims loopmax tfopt_lim tfoptmax_stop

set loopmax 100

set tfoptmax_stop 12

set tfopt_max 0.0

set loopcnt 0

set file_log [open $par(name)\.max w]

set s_tfopt "NO"

puts $file_log "loopmax is $loopmax"

puts $file_log "tfoptmax_stop is $tfoptmax_stop"

while {$tfopt_max < $tfoptmax_stop && $loopcnt < $loopmax} {

incr loopcnt

puts "$loopcnt"

set lims [get_lims $par(SWH) $par(cp)]

set rfsh [rand_shape 100 $par(NOC) [expr round($par(NOC)*0.2)] ]

set tfopt [oc_optimize $rfsh -max 100 -min 0]

if {$tfopt > $tfopt_max} {

set tfopt_max $tfopt

puts $file_log "new max tfopt is $tfopt_max"

save_shape $rfsh $par(name)\_sol_$tfopt\_rf.dat

puts $file_log "rf field is saved"

} else {

puts $file_log "no good rf field is found for loop no. $loopcnt"

}

free_all_shapes

}

close $file_log

}

**Reference**

1. Hors, P. J. A new method for water suppression in the proton NMR spectra of aqueous solutions. *J. Magn. Reson.* **54**, 539-542, (1983).

2 Ogg, R. J., Kingsley, R. & Taylor, J. S. WET, a T_1_-and B_1_-insensitive water-suppression method for in vivo localized ^1^H NMR spectroscopy. *J. Magn. Reson.* **104**, 1-10, (1994).

3 Bottomley, P. A. Spatial localization in NMR Spectroscopy in vivo. *Ann. N. Y. Acad. Sci.* **508**, 333-348, (1987).
